# Supplementary material for: Smart 3D super-resolution microscopy reveals the architecture of the RNA scaffold in a nuclear body
Source: Nat Commun. 2025 Nov 27;16:10689. doi: 10.1038/s41467-025-65723-x (PMC12660752; doi:10.1038/s41467-025-65723-x)
Supplement: Supplementary file 1 — Supplementary Information [file 41467_2025_65723_MOESM1_ESM.pdf]

Supplementary Information

Smart 3D super-resolution microscopy reveals the architecture of the RNA scaffold in a nuclear body

Enya S. Berrevoets*[1], Laurell F. Kessler*[2], Ashwin Balakrishnan[2], Ellen Kazumi Okuda[3,4], Michaela Müller-McNicoll[3,5], Bernd Rieger#[1], Sjoerd Stallinga#[1], Mike Heilemann#[2]

[1]*Department of Imaging Physics, Delft University of Technology, Delft, The Netherlands*

[2]*Institute of Physical and Theoretical Chemistry, Goethe University, Frankfurt am Main, Germany*

[3]*Institute of Molecular Biosciences, Goethe University, Frankfurt am Main, Germany*

[4]*IMPRS on Cellular Biophysics, Frankfurt am Main, Germany*

[5]*Max Planck Institute for Biophysics, Frankfurt am Main, Germany*

* Equal contribution

# Corresponding authors

Correspondence: b.rieger@tudelft.nl, s.stallinga@tudelft.nl, heilemann@chemie.uni-frankfurt.de

This PDF file includes:

Supplementary Figures 1 - 13

Supplementary Tables 1 - 11

Supplementary Figures

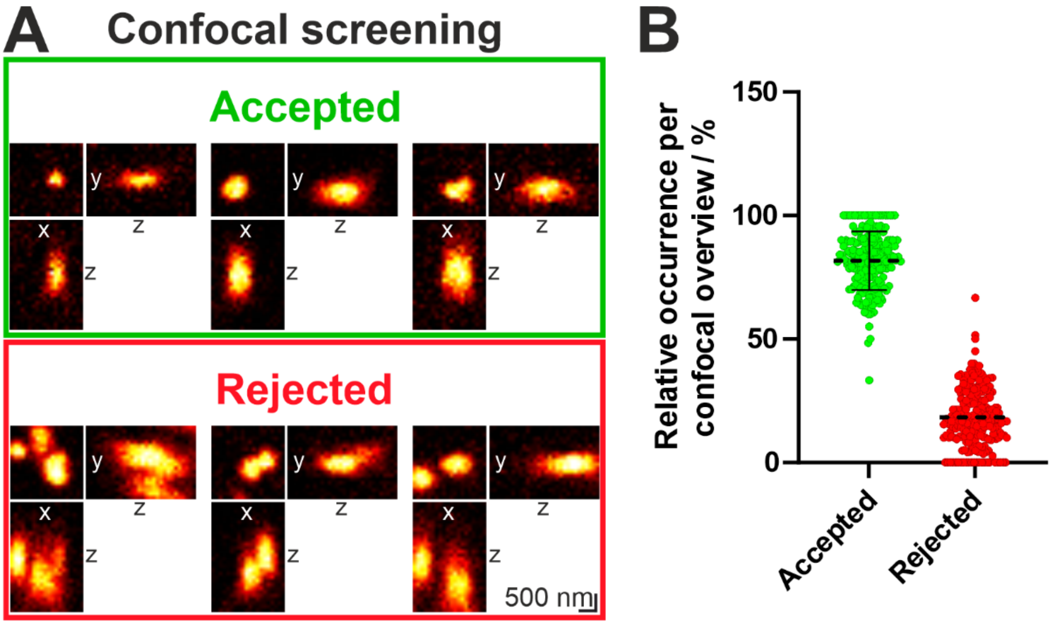

Supplementary Figure 1. Selection criteria for high-resolution confocal screening of paraspeckles. A Examples of confocal ROIs containing paraspeckles. ROIs matching with a single ellipsoidal template are accepted for a subsequent STED measurement (green). ROIs containing multiple paraspeckles are rejected (red). Scale bar = 500 nm. **B** Fractions of automatically found paraspeckles that are measured with STED and further analyzed (green) and multiple paraspeckles packed densely that were not measured with STED (red) per screened confocal overview. Displayed are mean and standard error. N = 215.

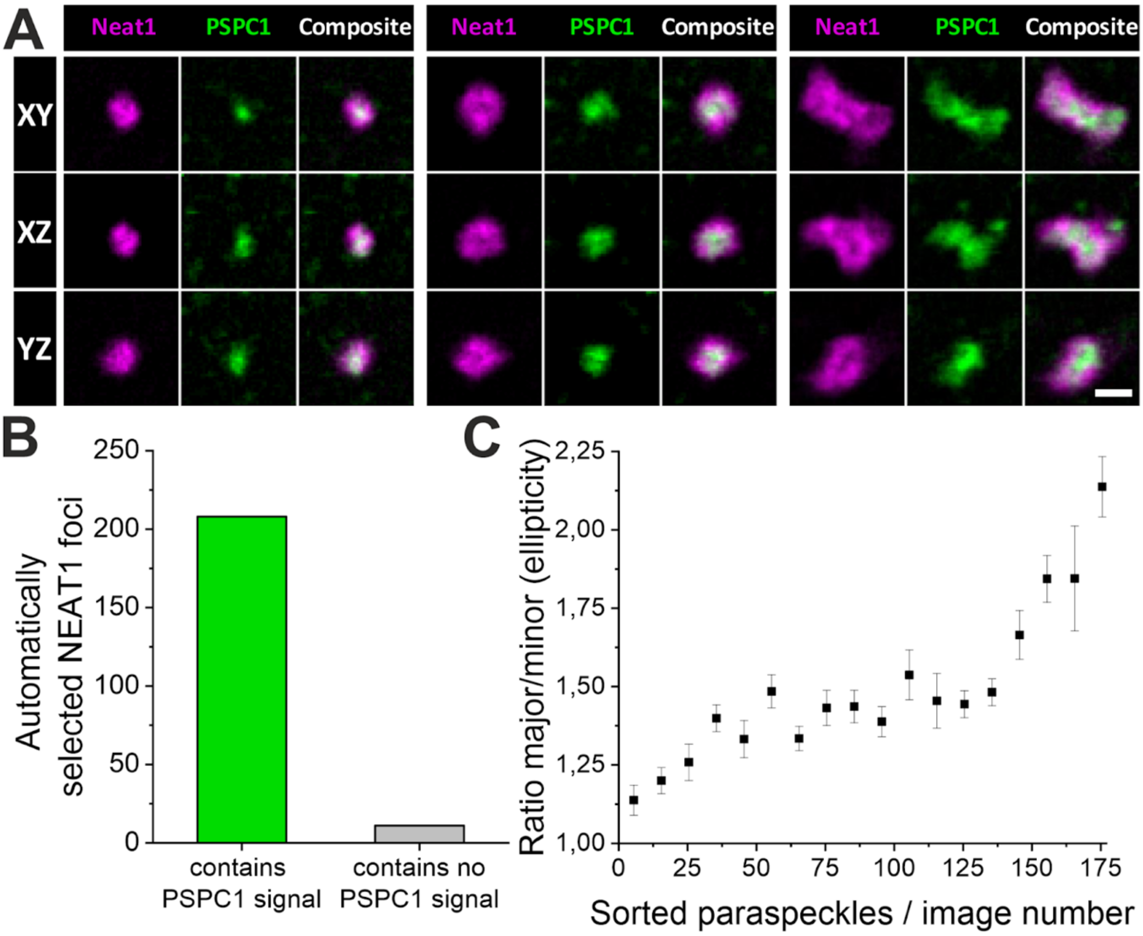

Supplementary Figure 2. Validation of the automated super-resolution imaging and analysis workflow.

A Examples of automatically selected *NEAT1* foci. Shown is signal of RNA-FISH probes binding to the very 3' and 5' end of *NEAT1_2* (magenta; reference), immunofluorescence signal of PSPC1-GFP (green) and composites of both signals. Scale bar = 500 nm. **B** Number of automatically selected and measured particles showing overlap with PSPC1-GFP immunofluorescence signal and with no overlap. [N = 219 (208 showed PSPC1 signal)]. **C** Ratio of the paraspeckles' major-to-minor shell-to-center distance, obtained from paraspeckles showing overlap with PSPC1 signal. In each instance, a total of 10 data points were summarized. Error bars represent the standard error of the mean (s.e.m.).

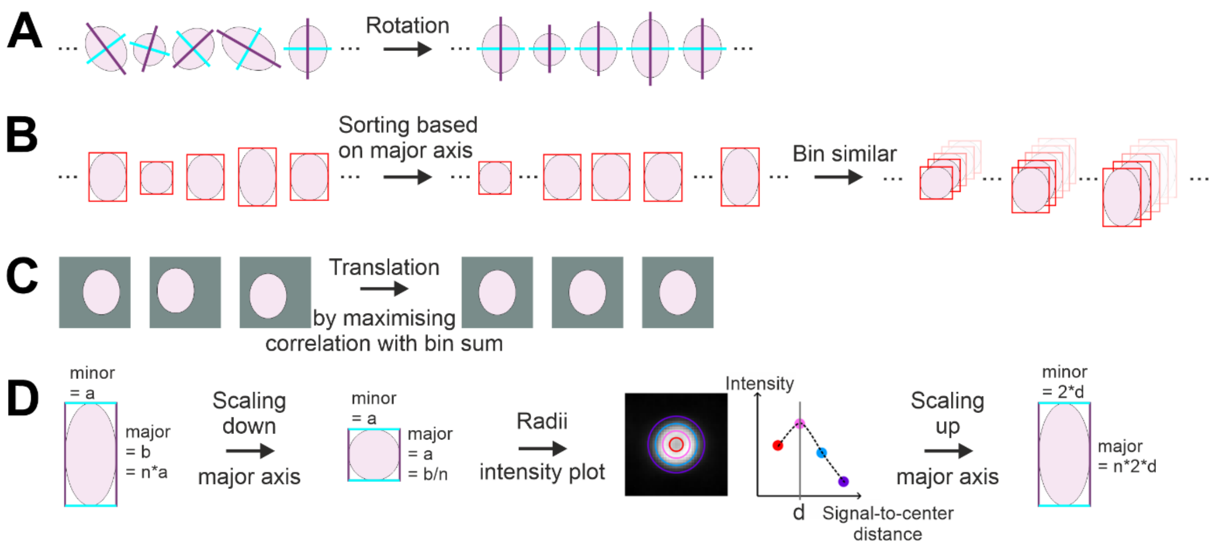

Supplementary Figure 3. Schematics of the image processing steps. 2D schematic depictions of the different processing steps after the image acquisition. In reality, the images are 3D, but the same principles apply. **A** The raw images are rotated to align the paraspeckle's major axis with the vertical image axis (**Methods**). **B** The rotationally aligned images are sorted based on their major axis length, obtained using a bounding box (**Methods**). Images with a similar major axis length are binned together. **C** Images within each bin are translated to maximize their cross-correlation with the sum over all images in that bin. **D** The major axis of each image is then scaled down to match its minor axis and a radial intensity plot is used to determine the average signal-to-center distance of the paraspeckle along the minor axis. The major axis length is then determined by multiplying this minor axis length with the major/minor axis scaling factor. A more detailed explanation of image processing is given in the **methods**.

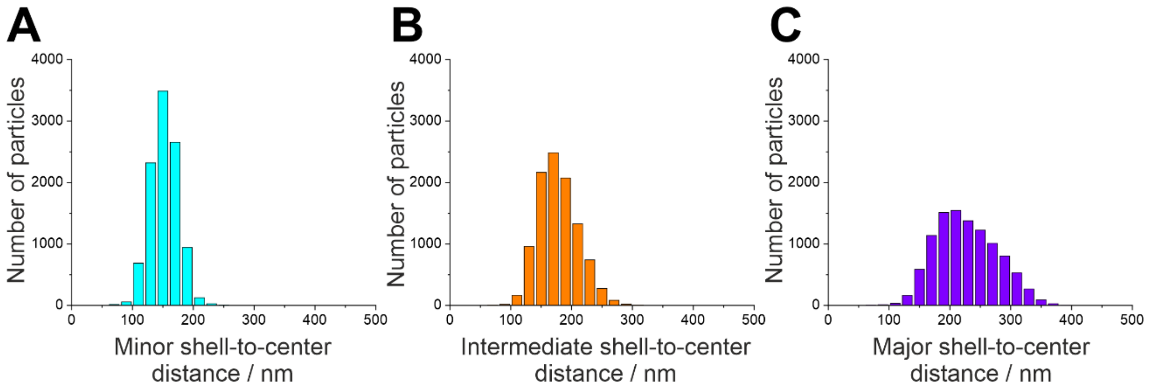

Supplementary Figure 4. Distribution of the paraspeckle lengths along the three axes. Frequency histograms of paraspeckle lengths along their **A** minor **B** intermediate and **C** major shell-to-center distances. N=13,801 paraspeckle containing volumes from 34 independent experiments (**Supplementary Table 3**).

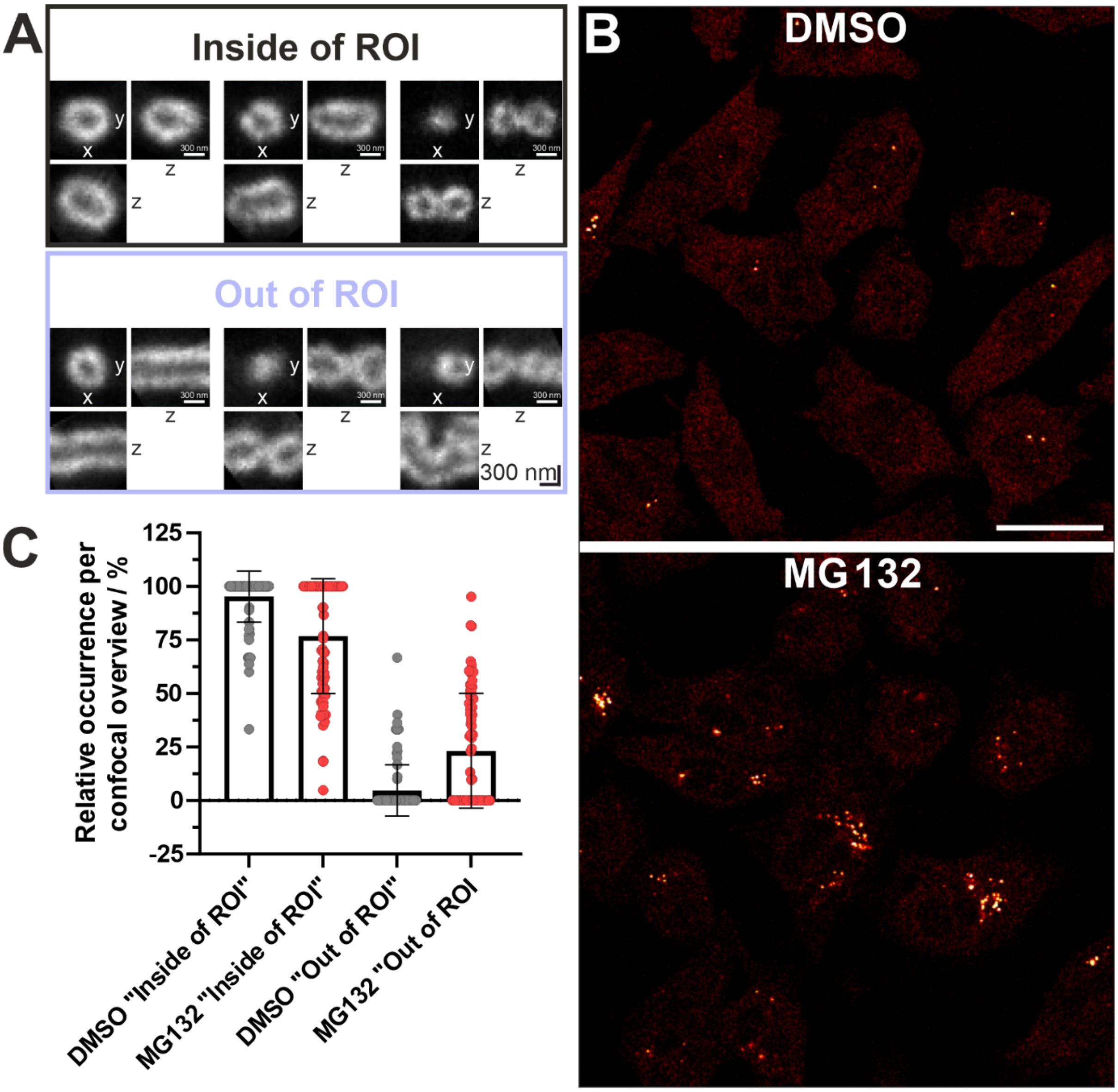

Supplementary Figure 5. Exemplary paraspeckles confined within or extend beyond the ROI acquired with volumetric STED. A Shown is signal of RNA-FISH probes binding to the very 3’ and 5’ end of *NEAT1_2.* ROIs containing paraspeckles protruding outside the STED volume are categorized as Out-of-ROI. Scale bar = 300 nm. **B** Exemplary confocal measurements of HeLa cells treated with DMSO (left) and MG132 (right). Cells were treated with 10 µM of MG132 (M7449-200UL, Sigma Aldrich) or the same volume of DMSO for 4 hours, fixed and stained using RNA-FISH probes hybridizing to the 3’ end and 5’ end of *NEAT1_2*. Scale bar = 20 µm. **C** Fractions of automatically found paraspeckles that are measured with STED and that are inside or outside an ROI for cells treated with MG132 (light red) and the control DMSO (gray). Displayed are mean values and standard errors. N = 111 (DMSO), N = 76 (MG132).

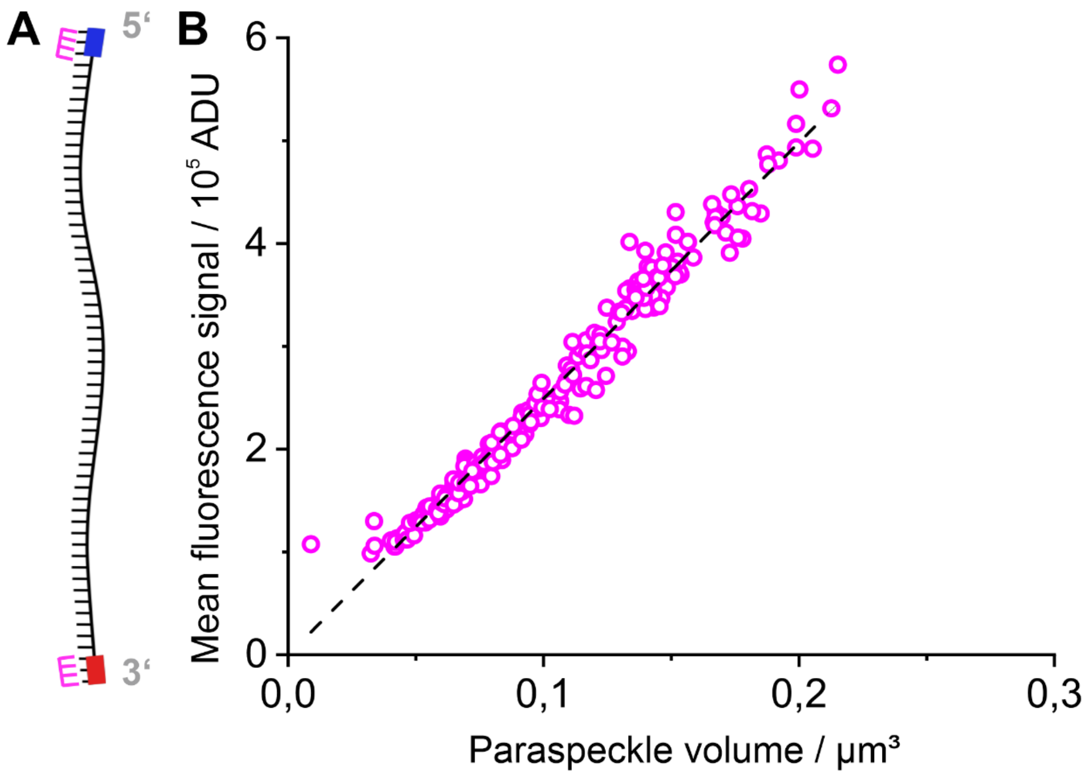

Supplementary Figure 6. Fluorescence intensity of reference probes scales with Volume. (**A**) Scheme of RNA-FISH staining at the shell reference positions at the ends of *NEAT1_2*. (**B**) Total fluorescence intensity plotted as a function of paraspeckle volume for the shell reference. Each point represents one bin of similar paraspeckle images (see **S1B**) and is calculated by averaging the summed image intensities in that bin. N=13,801 paraspeckle containing volumes from 34 independent experiments (**Supplementary Table 3**). Fit results for linear regression (black dotted line) of **B** is given in **Supplementary Table 5**.

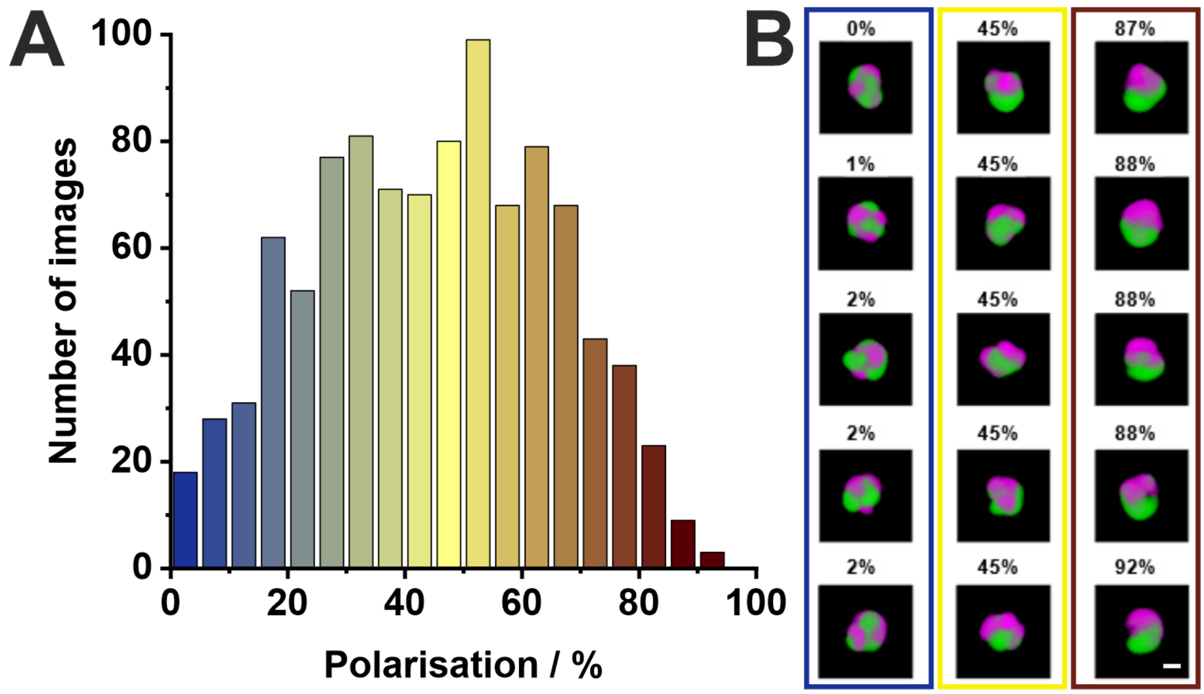

Supplementary Figure 7. Polarization degrees for simulated paraspeckles with random distributed 3' and 5' ends of *NEAT1_2.* **A** Distribution of measured degree of polarization for 1000 simulated paraspeckles. **B** Examples of simulated spherical paraspeckles with the 5' and 3' ends of *NEAT1_2* randomly distributed across the paraspeckle shell region (**Methods**).

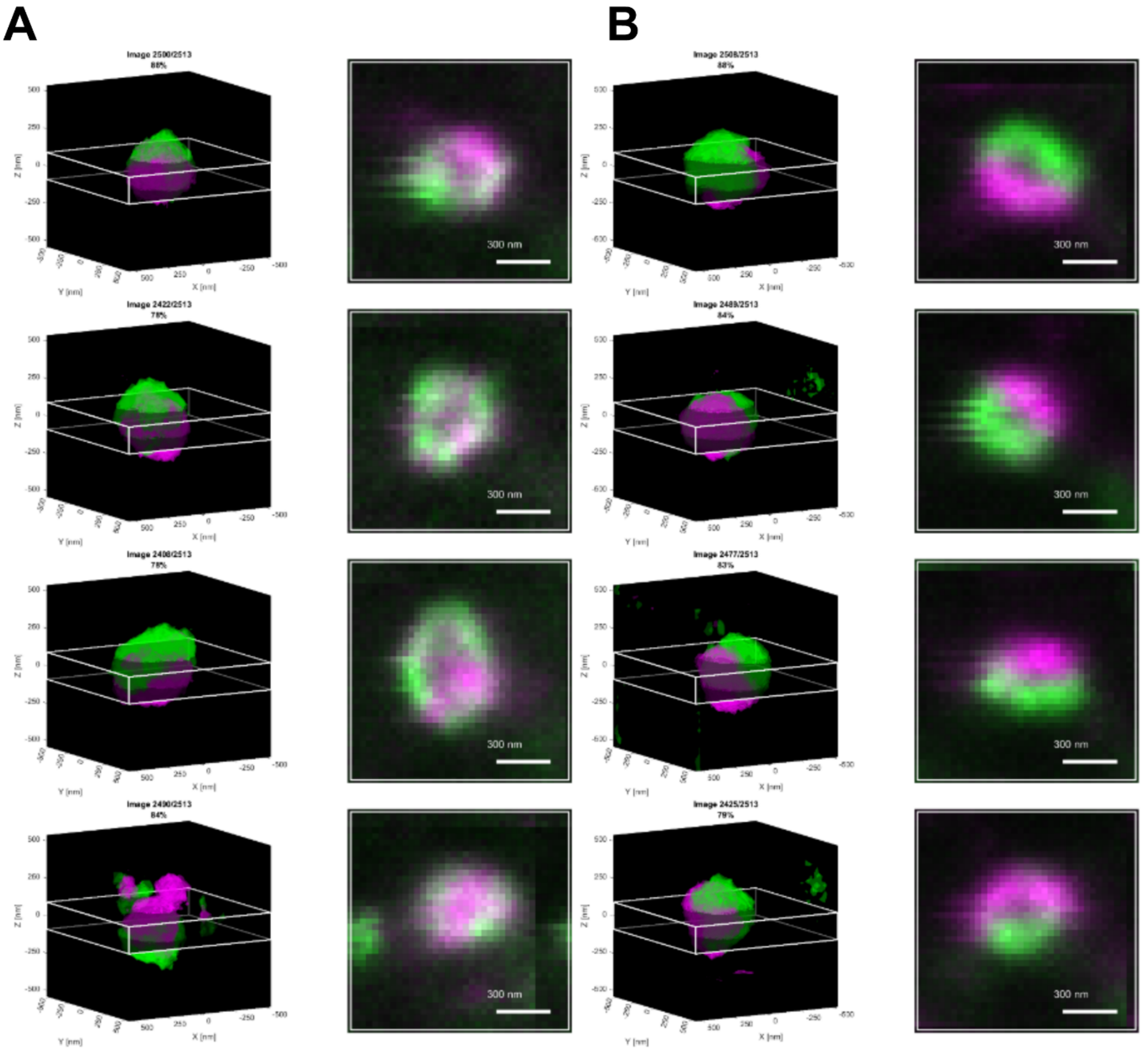

Supplementary Figure 8. Representative isosurface plots and corresponding 2D projections of paraspeckle volumes with different polarizations. Selected paraspeckles with a degree of polarization above 78%**. A** Four examples of paraspeckles for which their polar nature is not apparent from the 2D projections. **B** Four examples of paraspeckles for which their polar nature is identifiable in 2D as well as in 3D. 2D projections correspond to 200 nm thick slices; Scale bar: 300 nm. Green: 5' signal; magenta: 3' signal.

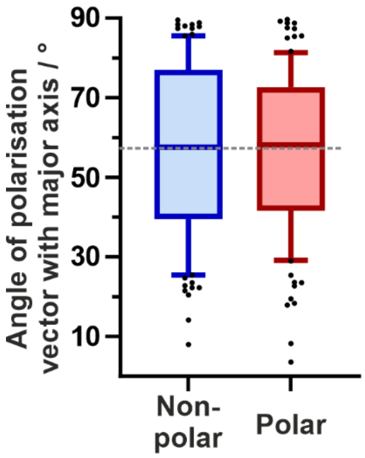

Supplementary Figure 9. Angle of polarization vector relative to the major axis for nearly spherical paraspeckles. Angle for spherical paraspeckles (elongation ≤1.3), grouped into non-polar (100 paraspeckles with lowest polarization, 3 independent experiments, blue) and polar (100 paraspeckles with highest polarization, 3 independent experiments, red). The horizontal gray dotted line indicates the random angle of 57°. The line inside the box indicates the median, the box indicates 25th and 75th percentile and the whiskers indicate 10th and 90th percentile. The points indicate outliers.

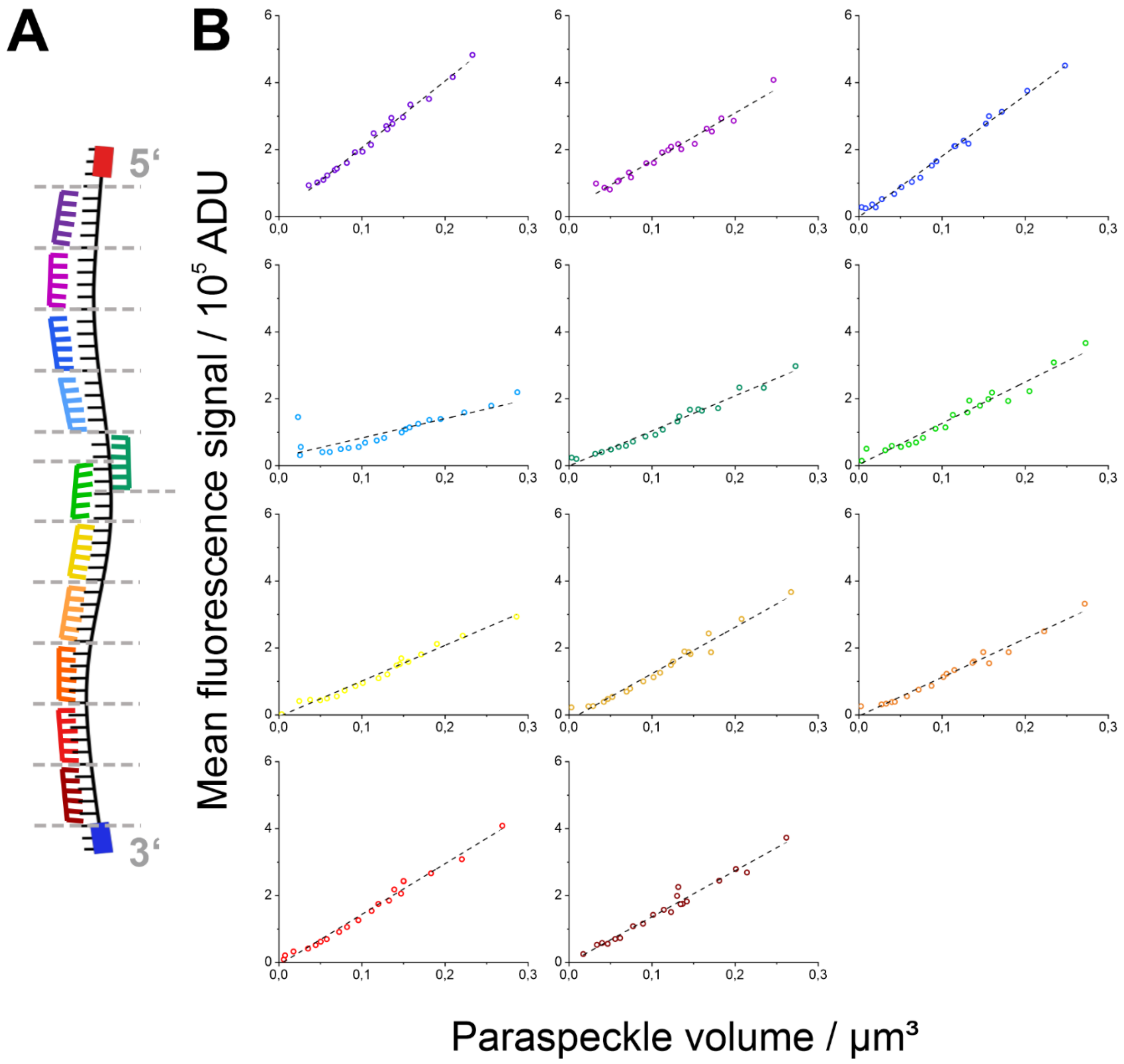

Supplementary Figure 10. Fluorescence intensity of internal probes scales with Volume. (**A**) Scheme of RNA-FISH staining at the different 2K nt regions along the *NEAT1_2* RNA. (**B**) Total fluorescence intensity plotted as a function of paraspeckle volume for the different regions along *NEAT1_2*. Each point represents one bin of similar paraspeckle images (see **S1B**) and is calculated by averaging the summed image intensities in that bin. N=967-2992 paraspeckle containing volumes from up to 4 independent experiments were performed per *NEAT1_2* region (**Supplementary Table 3**). Fit results for linear regression (black dotted lines) of **B** is given in **Supplementary Table 5**.

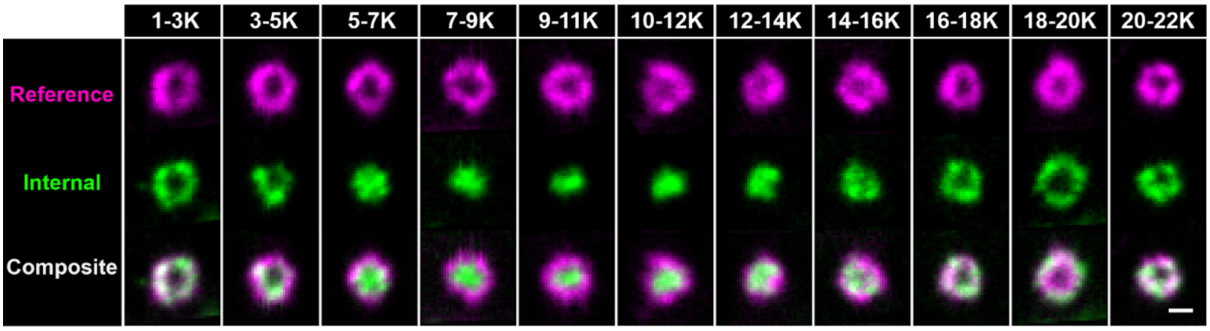

Supplementary Figure 11. Representative 2D cross-sections of individual spherical paraspeckles from all 11 RNA-FISH probe sets. For visualization, all two-color 3D STED volumes were aligned rotationally and translationally using a transformation matrix calculated from reference channel signal. Subsequently, images are obtained by summing up the three center XY layers. The top row shows fluorescence images of RNA-FISH probes hybridizing to the 3' and 5' end of *NEAT1_2* (reference; magenta). The middle row shows fluorescence images of RNA-FISH probes, each hybridizing to the corresponding internal position of NEAT1_2 (Internal; green). The bottom row shows the composite of reference and internal probes. Scale bar = 300 nm.

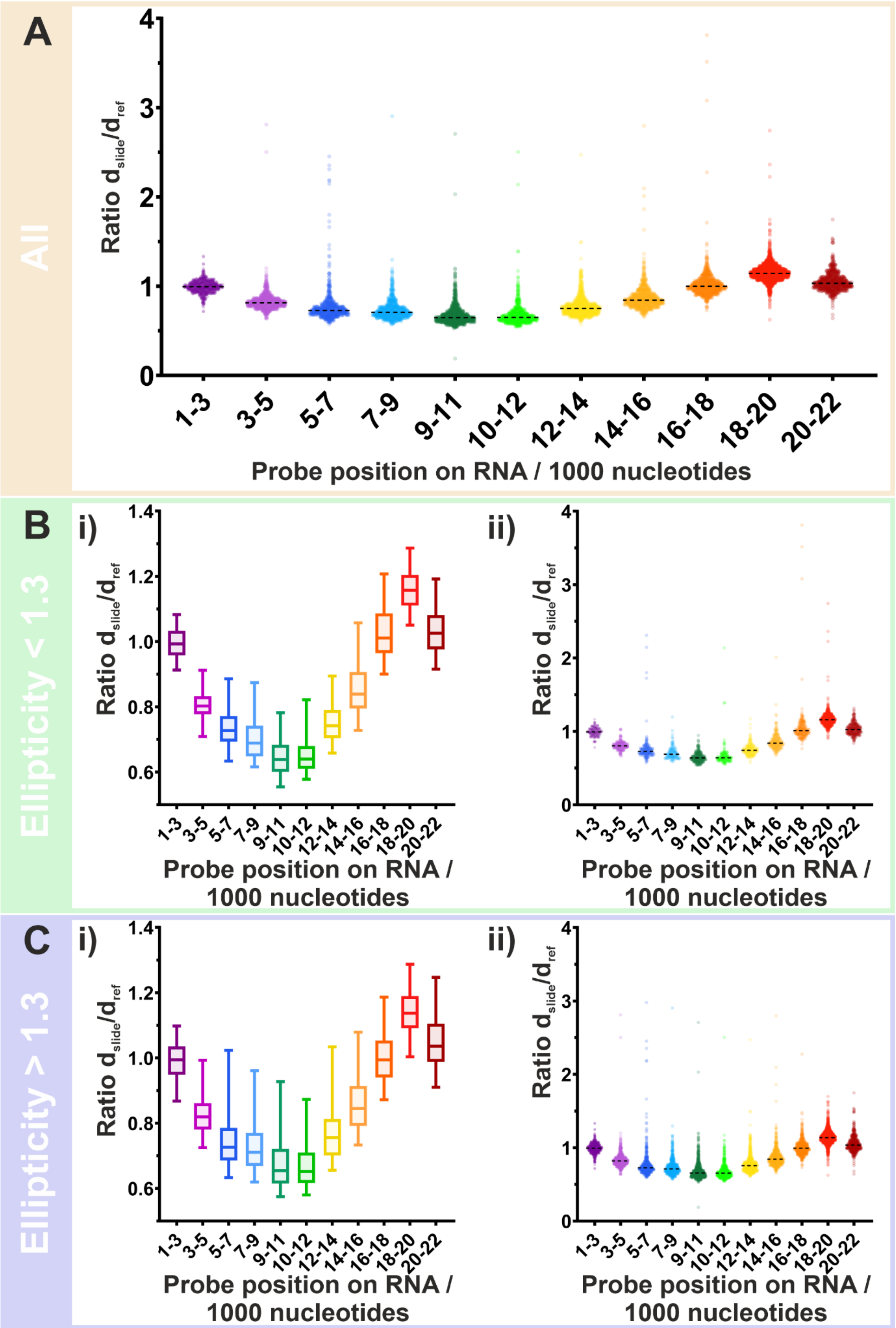

A
All
Ratio d_{slide}/d_{ref}
Probe position on RNA / 1000 nucleotides
1-3
3-5
5-7
7-9
9-11
10-12
12-14
14-16
16-18
18-20
20-22
B
Ellipticity < 1.3
i)
ii)
C
Ellipticity > 1.3

Supplementary Figure 12. Distance-to-center ratios for 11 different internal/reference RNA-FISH probe sets.

A The average distance of the 11 different *NEAT1_2* sections to the paraspeckle center, relative to the to-center distance of the combined 5'/3' reference positions, as calculated from the individual images' radial intensity distribution including all data points. Dotted line indicates median. **B** Distance-to-center ratios for spherical paraspeckles (ellipticity ≤ 1.3) i) as box & whiskers (median, 1st&3rd quartile, 5-95 percentile) ii) including all data points; dotted line indicates median. **C** Distance-to-center ratios for ellipsoidal (ellipticity > 1.3) i) as box & whiskers (median, 1st&3rd quartile, 5-95 percentile) ii) including all data points; dotted line indicates median. N = 967 to 2,992 paraspeckle containing volumes from up to 4 independent experiments were performed per *NEAT1_2* region (**Supplementary Table 3**).

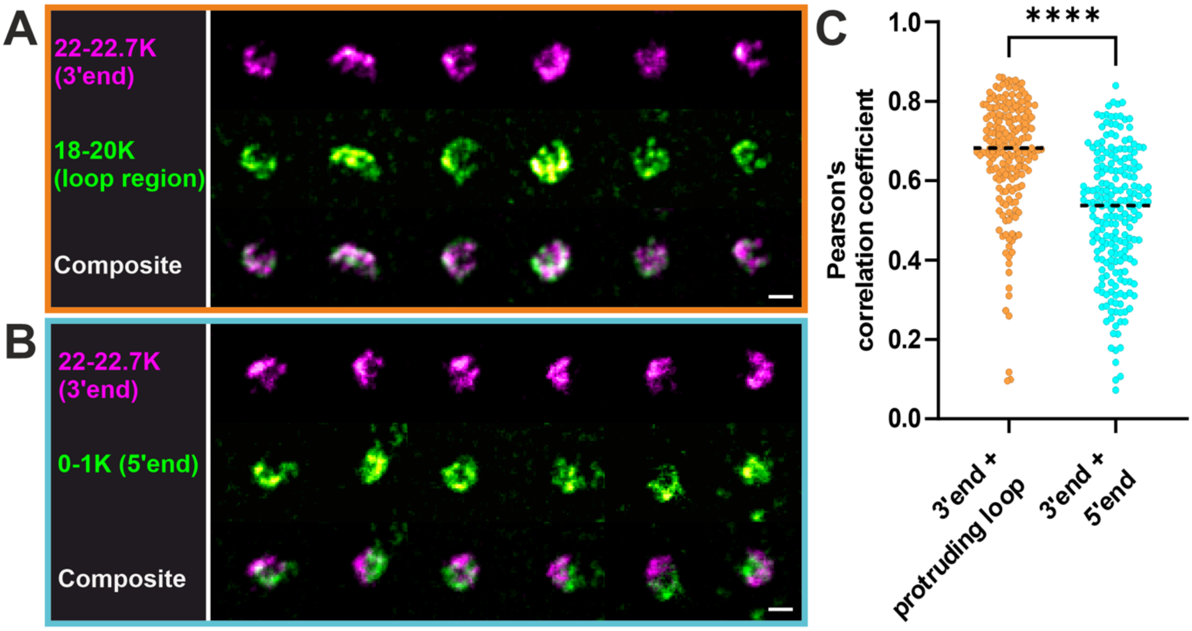

Supplementary Figure 13: Exemplary two-colour STED measurements of 3'loop and 3' end, as well as 3' end and 5' end of *NEAT1_2*. A Selected images showing potentially polarized paraspeckles labelled with FISH probes targeting the protruding 3'loop (18-20K nt) and the 3' end (22-22.7K nt). Scale bar = 300 nm. **B** Selected images showing potentially polarized paraspeckles labelled with FISH probes targeting the 5' end (0-1K nt) and the 3' end (22-22.7K nt). Scale bar = 300 nm. **C** Calculated Pearson correlation coefficient for the image sets shown in (A) and (B). Black dotted line indicates the median. Significance was tested using a Mann-Whitney U test (****: $p<0.0001$). N = 199 (3' end and 3'loop), N = 208 (3' end and 5' end). Mean and standard error are in **Supplementary Table 10**.

Supplementary Tables

Supplementary Table 1: Imager and docking strand sequences used in this study. Modified LNA nucleotides are colored blue[1]. Docking sequences were synthesized together with the FISH targeting sequences from 5' to 3' end.

Name	Sequence	Supplier
P2-STAR 635P	5' TATG**TAG**ATAA-STAR 635P-3'	biomers.net
P3-STAR 580	5' TAA**TGA**AGAAA -STAR 580-3'	biomers.net
P2 docking strand (FISH probe)	5' *FISH probe sequence*-TTATCTACATA-3'	Eurofins Genomics
P3 docking strand (FISH probe)	5' *FISH probe sequence*-TTTCTTCATTA-3'	Eurofins Genomics

Supplementary Table 2: Median Resolution of measured STED images. Resolutions were determined by performing single image Fourier ring correlation [2] on 100 central cross sections (sum of 3 central slices) of images acquired using P3-STAR 580 and P3-STAR 635P. Errors are given as standard deviation.

Imager strand	Cross section view	Resolution / nm
P3-STAR 580	XZ	133±15
	XY	112±13
	YZ	123±15
P3-STAR 635P	XZ	109±13
	XY	100±16
	YZ	106±12

Supplementary Table 3: FISH staining schemes and number of recorded volumes used for figures presented in this study. For the respective figure, the corresponding section is indicated which was marked, followed by the connected docking strand. For the microscopy measurement, the corresponding imager strand was added to the imaging buffer.

Experiment (figure)	Staining scheme of nucleotide position (magenta = P3-STAR 580; green = P2-STAR 635P)	Number of images recorded	Number of independent measurements
Figure 2B	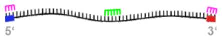 Shell(0-1K; 22-22.7K)-P3 Core(10-12K)-P2	1	1
Figure 3; Supplementary Figure 1, 4, 5A, 6	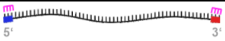 Reference(0-1K; 22-22.7K)-P3	13801	34
Figure 4; Supplementary Figure 9	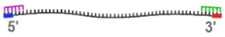 5prime(0-4K)-P3 3prime(16-22K)-P2	2513	3
Figure 5; Supplementary Figure 10, 11, 12	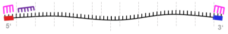 Reference(0-1K; 22-22.7K)-P3 Sliding(1-3K)-P2	1095	3
Figure 5; Supplementary Figure 10, 11, 12	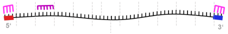 Reference(0-1K; 22-22.7K)-P3 Sliding(3-5K)-P2	1326	4
Figure 5; Supplementary Figure 10, 11, 12	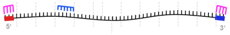 Reference(0-1K; 22-22.7K)-P3 Sliding(5-7K)-P2	973	3
Figure 5; Supplementary Figure 10, 11, 12	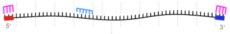 Reference(0-1K; 22-22.7K)-P3 Sliding(7-9K)-P2	967	3
Figure 5; Supplementary Figure 10, 11, 12	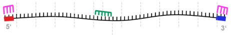 Reference(0-1K; 22-22.7K)-P3 Sliding(9-11K)-P2	1335	2
Figure 5; Supplementary Figure 10, 11, 12	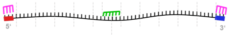 Reference(0-1K; 22-22.7K)-P3 Sliding(10-12K)-P2	1029	3
Figure 5; Supplementary Figure 10, 11, 12	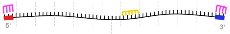 Reference(0-1K; 22-22.7K)-P3 Sliding(12-14K)-P2	1048	1
Figure 5; Supplementary Figure 10, 11, 12	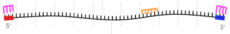 Reference(0-1K; 22-22.7K)-P3 Sliding(14-16K)-P2	1020	3
Figure 5; Supplementary Figure 10, 11, 12	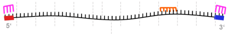 Reference(0-1K; 22-22.7K)-P3 Sliding(16-18K)-P2	1018	3
Figure 5; Supplementary Figure 10, 11, 12	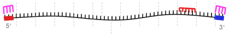 Reference(0-1K; 22-22.7K)-P3 Sliding(18-20K)-P2	2992	3
Figure 5; Supplementary Figure 10, 11, 12	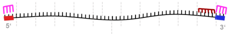 Reference(0-1K; 22-22.7K)-P3 Sliding(20-22K)-P2	999	3

Supplementary Figure 2	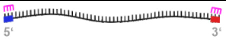 Reference(0-1K; 22-22.7K)-P3 GFP-PSPC1 NB@GFP-STAR 635P	219	3
Supplementary Figure 13	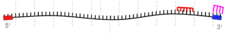 3’ end (22-22.7K)-P3 Sliding(18-20K)-P2	199	3
Supplementary Figure 5B,C	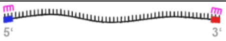 Reference(0-1K; 22-22.7K)-P3	111 (DMSO) 76 (MG132)	3 (DMSO) 5 (MG132)
Supplementary Figure 13	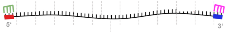 3’ end (22-22.7K)-P3 5’ end (0-1K)-P2	208	3

Supplementary Table 4: Shell-to-center distances of paraspeckles measured from single-color STED measurements. The errors given are the s.e.m.

Ellipticity	Major axis / nm	Intermediate axis / nm	Minor axis / nm
1.14 ± 0.9	126 ± 18	133 ± 21	114 ± 18
1.30 ± 0.15	187 ± 11	163 ± 20	146 ± 14
1.49 ± 0.14	234 ± 10	186 ± 23	160 ± 16
2.0 ± 0.3	315 ± 25	215 ± 32	162 ± 16

Supplementary Table 5: Values for linear regression of fluorescence intensities measured for targeted nucleotide positions of NEAT1_2 RNA. Errors are given as standard error.

Target nucleotide position (nucleotides form 5’ to 3’) / 1000 nucleotides	Slope / 10^5*ADU/µm^3	Intersection with the Y-axis / 10^5*ADU	R^2
0-1 and 22-22.7	24.9 ± 0.3	-0.01 ± 0.04	0.9746
1-3	19.9 ± 0.5	0.07 ± 0.06	0.99067
3-5	14.4 ± 0.6	0.22 ± 0.08	0.96993
5-7	18.1 ± 0.4	-0.01 ± 0.05	0.99273
7-9	5.7 ± 0.9	0.30 ± 0.14	0.70014
9-11	10.5 ± 0.4	-0.01 ± 0.05	0.9780
10-12	12.3 ± 0.7	0.03 ± 0.09	0.95826
12-14	10.6 ± 0.4	-0.05 ± 0.05	0.98243
14-16	13.9 ± 0.6	-0.15 ± 0.07	0.97511
16-18	11.6 ± 0.5	-0.03 ± 0.06	0.97736
18-20	15.1± 0.5	-0.07 ± 0.06	0.98644

20-22	13.8 ± 0.6	-0.01 ± 0.08	0.97211

Supplementary Table 6: Degree of polarization (P) of paraspeckles measured from two-color STED measurements. The errors given are the s.e.m.

Ellipticity	Degree of Polarization (P) / %
≤1.3	44.1 ± 0.8
>1.3	39.8 ± 0.6

Supplementary Table 7: Angle between polarization vector and major axis (α) of paraspeckles measured from two-color STED measurements. The errors given are the s.e.m.

Ellipticity	Degree of Polarization (P) / %	Angle between polarization vector and major axis (α) / °
≤1.3	<14.7	57 ± 3
≤1.3	>72.0	57 ± 3
>1.3	<8.3	59 ± 3
>1.3	>74.1	72 ± 2

Supplementary Table 8: Bin sizes used to generate 2D cross-sections of averaged spherical paraspeckles.

Target nucleotide position (nucleotides form 5’ to 3’) / 1000 nucleotides	1-3	3-5	5-7	7-9	9-11	10-12	12-14	14-16	16-18	18-20	20-22
Bin sizes	75	99	93	67	107	76	92	69	89	274	61

Supplementary Table 9: Ratio of internal to reference distance to the center (d) for the 11 different internal probes measured.

Target nucleotide position (nucleotides form 5’ to 3’) / 1000 nucleotides	Ellipticity	Median d(internal)/d(reference)	5% Percentile d(internal)/d(reference)	95% Percentile d(internal)/d(reference)
1-3	all	0.9943	0.8713	1.093
3-5	all	0.8137	0.7248	0.9811
5-7	all	0.7270	0.6333	1.005
7-9	all	0.7076	0.6195	0.9516
9-11	all	0.6474	0.5704	0.8863

10-12	all	0.6500	0.5800	0.8541
12-14	all	0.7502	0.6569	1.011
14-16	all	0.8435	0.7327	1.073
16-18	all	0.9980	0.8829	1.189
18-20	all	1.143	1.019	1.286
20-22	all	1.033	0.9142	1.233
1-3	≤1.3	0.9927	0.9129	1.083
3-5	≤1.3	0.8024	0.7090	0.9121
5-7	≤1.3	0.7275	0.6336	0.8860
7-9	≤1.3	0.6890	0.6159	0.8741
9-11	≤1.3	0.6387	0.5548	0.7818
10-12	≤1.3	0.6397	0.5779	0.8217
12-14	≤1.3	0.7419	0.6585	0.8944
14-16	≤1.3	0.8387	0.7279	1.057
16-18	≤1.3	1.011	0.9004	1.207
18-20	≤1.3	1.157	1.050	1.286
20-22	≤1.3	1.026	0.9154	1.192
1-3	>1.3	0.9943	0.8677	1.098
3-5	>1.3	0.8192	0.7251	0.9928
5-7	>1.3	0.7264	0.6333	1.023
7-9	>1.3	0.7108	0.6198	0.9608
9-11	>1.3	0.6547	0.5746	0.9273
10-12	>1.3	0.6521	0.5801	0.8729
12-14	>1.3	0.7560	0.6555	1.034
14-16	>1.3	0.8450	0.7331	1.079
16-18	>1.3	0.9939	0.8719	1.186
18-20	>1.3	1.137	1.004	1.287
20-22	>1.3	1.036	0.9101	1.247

Supplementary Table 10: Calculated Pearson correlation coefficient between image pairs of paraspeckles stained with FISH probes targeting the 3' loop (18-20K) and 3' end (22-22.7K nt), and the 3' end (22-22.7K nt) and 5' end (0-1K nt). Given are mean and standard error values.

Image pair	Pearson correlation coefficient
3' end + 3' loop (18-20K)	0.51 ± 0.17
3' end + 5' end	0.66 ± 0.15

Supplementary Table 11: List of FISH probes used to stain Neat1_2 RNA in this study.

Target nucleotide position (nucleotides form 5' to 3')	Seqences (5' to 3')
Reference (0-1K; 22-22.7K)	tgcggatattttccatgcag<br>ctcgctcagctatgcaagag<br>caagttgaagattagccctc<br>agcccttggtctggaaaaaa<br>aagttcagttccacaagacc<br>caggccgagcgaaaattaca<br>ttaactccacatcactcctc<br>ctgtcaaacatgctaggtgc<br>aagcgttggtcaatgttgtc<br>aaaaggagcactgccacctg<br>gtggagtgagctcacaagaa<br>cttaccagatgaccaggtaa<br>acaataccgactccaacagc<br><br>actgccatcactcgaaatga<br>tccattctctgatctgtcag<br>agtggaaatggttctctggg<br>ctaagacctgtggacacttc<br>tcttttcatctgaacaggga<br>tttttgactctatgcagatc<br>cttgcattttatacccatgg<br>gaggacatctctgtgtagta<br>atttccagagtgagtctagg<br>acgagcagaggcacaagctt<br>cacaggtgagaaggtgctgg<br>aactgaacacgaggcacagt<br>ccaaggagcatgaagtcaga<br>cagcttcacatccacatgtc<br>taaaggcatagccaggggac<br>aaaagaaacacctgcggcgg<br>aaaacctgagtgcggccatg<br>ttcacagatgggaaggtgaa<br>tttgctttttcctgctcact
1-3K	tcctcttactagaatgccaa<br>actgtatctctaaccaaccc<br>aattcccttcaacctgcatt

	gcaacaacttaaccaacact<br>ctaagcaacttctcacttcc<br>taacacttcttcagtcttcc<br>actctgtgtcacctgttttc<br>cctttggttctcggaaaact<br>tgtgagatggcatcacacac<br>ccaggaggaagctggtaaag<br>ctctgaaacaggctgtcttg<br>cacttgataacacccacacc<br>cccatctttcaagtgactaa<br>cagcgaaggatgctgatctg<br>aaccacctaagttgctaagg<br>cacaccttcacagtttcaaa<br>gtggtcccttaaatacgtta<br>agaagagcccatctaatctc<br>acacctgtgacaaatgagga<br>gatgtgtttctaaggcacga<br>tgccattattaatatcgacc
3-5K	tacagtgaccacaaaaggtt<br>gcaaaggtacatggattctg<br>attttaggtgatagtttccc<br>tttttaggtcttgttttcca<br>catgtagtaaaggcacctcg<br>ccattggtattactttacca<br>ggttaagaactgaactacca<br>ttgtttgcatcatccccaag<br>ctctaaatcccaacgacagt<br>cccatacatgcgtgactaat<br>atttcacaacagcatacccg<br>ccagtactttcaaccatcta<br>agttcttaccatacagagca<br>ttgtgctgtaaaggggaaga<br>tgataggggtcgagaaatgt<br>ccaaagtcgttatgaaggca<br>ggagctagcaaatctagacc<br>tcctacatggccttaattac<br>ttctatctgtggttaccatg<br>gcagaccacttgggataata<br>accaactctcaacagagagc<br>cttgagtggtttcatcacat<br><br>aaagtactccccacctacac<br>accacttagaactctaacca<br>cacttagacccaaatcccag<br>agtgaggtaagaattacgca<br>aagcccagacgttttagtca<br>ttactgtagcacgcacacag<br>caagatgtatcctccagatt<br>cagtgcaaggacagaacagt<br>cccaagaggtaacacagaac<br>tcctgtaagcaaaaccttac

	cattctacgcccaaagtttg<br>aaacaagcaccttccttctc<br>caggtgagtagaacaagcct<br>agacacagttgcaaggacgg<br>ggtcaccaagatataacggc<br>tttgtcagtagtgtacacgt<br>ccatgagcacagacagaagg<br>aaagaggaccctggattctg<br>cagggaaaggagttgcaagc<br>ataggaaaggaggcctggat<br>gagaaggcggttatagaggt<br>caggacgagaaacaaggagt
5-7K	cagggtggaagcaagagttt<br>ggcatctatagcagttatct<br>tcaattcttcttcagcatgc<br>caatccccaacatttatacc<br>tctgctcctgtatcaacaag<br>attggagtggaggcaaggag<br>tgaaggcaaagaatgtggcc<br>tctcacctgatctgaggata<br>taattgctggcactgccatc<br>aaggatgcaaacggattagc<br>gtctgaattcagatctatgc<br>cacacctctggaaattcaca<br>cccactcactgaattctatt<br>acttttgctattcttatgcc<br>actgaagagcctcaaccaat<br>tcatagtcagtcagatgcac<br>ctggcattaagggtccttgg<br>actgcacatggagaggttag<br>aagccagaggatgacacttc<br>tgcaccataatgagtaccac<br>tgttgccatttccaaacagt<br>agtattcattttgctatctt<br>tacctgttcatcttttcgta<br>gtcctaactagcaggctgaa<br><br>ggtaagattactgaggactt<br>ttccaaaagcaaatgtgccc<br>atgtacttatcccatgcata<br>tggcataatcccatttttgt<br>gacgtgaaaaagtctgagct<br>gtattcagcattcccaaaga<br>acagtcatttccttctcgaa<br>gcagtttagcaatatgaacc<br>tgaaaaaatccctccctgct<br>cctcaccagtgagaacacta<br>aagaggggaaggcatcaggt<br>tgtgataaagccggttctga<br>tcggaggagcaaaggttttc<br>gagggaaagcagactcgatc

	gacaaagcaatgctcacatc<br>tagagatgttcagtcaccag
7-9K	ccacggggaagaaggtttaa<br>ctcctcacacaaacacagat<br>caggaggtgggttctgagag<br>tgtgacaaccaccctcacag<br>gtcttctgaattgaaccctg<br>cattcaggaaacatcagcct<br>tcagggacaagcaacaacca<br>ctcagatggggaaatggaga<br>ctgccaaacatctacattca<br>agagctcaacaaggagtagc<br>agaggcaggagagttcactg<br>aggaagtggctagacctgac<br>cacagaaaagggagaggggc<br>tacatgaaaggtccagcgcc<br>gaatatacatgctgattccc<br>agtgtagaaacagaccagac<br>gccggtacagggaaatacta<br>agcctcttcaaatgtgtttg<br>ggctacatatcctttagtat<br>cagtttaacagctcaagaga<br>ctcacagctgcacgatttaa<br>aggggatacatgtgtacact<br>aaaccataagtgctcactgc<br>ttgagtctgtgaagagagtt<br>aagtgtagctttctggagtc<br><br>gttagtggctatgtagggaa<br>gaaagtagaatggggttgct<br>actgatatgaagtgcttgga<br>gccagtcacagacagacaaa<br>aggacactgtgctcagagaa<br>gctacaacatagacgcatct<br>gtccactaatggatgaatgg<br>gatgtgtgtacattacagca<br>gaactgaaggtaggatctca<br>tagtaataccacttctggct<br>actacagtggttcctcaata<br>aatggtgcagttgctatgaa<br>ttgtgcactcttggtgagaa<br>actcctacaagtcaacaaca
9-11K	tttcacatggttagtggtca<br>ccttcccaatgatactaaga<br>ggtttacaatcataagtggc<br>gtgctttttgcaccaacaat<br>ctgatttttaaagtcctggt<br>ggagtacatacatgtttagc<br>tggagaggagtgttcattgt<br>accaagacagtatttagtca<br>tattgactgacctggaacga

	atacacacacacacacacat<br>accccaaaagccattgaaat<br>tctatatgaccaaaagccat<br>tcagacttcactactgtaga<br>aggtgaccggtgcagtaaaa<br><br>ccacatattgggtacaatgt<br>gagggggcaaggtataaaa<br>agactcaaagtggggagggt<br>agagtgacataatggacact<br>gtgagagctaacttatgggt<br>ccaaatactgtgtgttctca<br>gtgaagcaactcaggaatgg<br>tggatggagctggaggatat<br>gttttttttttttttgcagc<br>aatgtggtatctctacacca<br>catcaaccagtgagtggata<br>attgcaaagatgtggaacca<br>agacacttgatggcagtaca<br>tgggtatctacccaaaggaa<br>atttgatctagcaatcccac<br>actggagcctaaaatttcca<br>gatcattacattccattgca<br>cctccatttaccagatttta<br>attcttgccttacaggagtg<br>tttgagttccaacagagaga<br>ttgagatttacccagttctg<br>tgtgtcctgaatcaaagatt<br>agcaagtaaaagagagccat<br>attcagaaatgcttaccaca<br>gtcctagtttttgtattgga<br>accataggagttactgtctg<br>agtggagaggagtgttcatt<br>gctatttcaatttcatcagt<br>atattgacagacctggaaca
10-12K	ccacatattgggtacaatgt<br>gagggggcaaggtataaaa<br>agactcaaagtggggagggt<br>agagtgacataatggacact<br>gtgagagctaacttatgggt<br>ccaaatactgtgtgttctca<br>gtgaagcaactcaggaatgg<br>tggatggagctggaggatat<br>gttttttttttttttgcagc<br>aatgtggtatctctacacca<br>catcaaccagtgagtggata<br>attgcaaagatgtggaacca<br>agacacttgatggcagtaca<br>tgggtatctacccaaaggaa<br>atttgatctagcaatcccac<br>actggagcctaaaatttcca

	gatcattacattccattgca<br>cctccatttaccagatttta<br>attcttgccttacaggagtg<br>tttgagttccaacagagaga<br>ttgagatttacccagttctg<br>tgtgtcctgaatcaaagatt<br>agcaagtaaaagagagccat<br>attcagaaatgcttaccaca<br>gtcctagtttttgtattgga<br>accataggagttactgtctg<br>agtggagaggagtgttcatt<br>gctatttcaatttcatcagt<br>atattgacagacctggaaca<br><br>atctcagacttcactactgt<br>ggaccacataagtggaaggt<br>atgattattaccttggccta<br>gcaagtgagagataaaccca<br>ttagatcagattcttttgca<br>gttccataagattacatgca<br>gctgaatcttaccatggtat<br>taaatgaactatcccctggg<br>tgagctctatccagtaaaca<br>ctaactgaggtatagtcaca<br>ccatggatgggagaactgat<br>aggacactgagaaacagtgt<br>aacacccacatatcacttct<br>ggaagtgaataaggcgaggt<br>ccatgcaacacgtttttcag
12-14K	tatatggagcgtcgtagagg<br>gatggcaactagcagaaact<br>actgcatacatgatagatca<br>ccaattacagtcttagtagg<br>gccatggacttacctaaatc<br>catccattacaatagcacat<br>aaattcatacactccacagc<br>catggcctttttctatagtg<br>gtccgggattatagaggaac<br>gttgtcattagtagttatct<br>catccacagccagaaaatgt<br>atgacatgttatagagccat<br>aacctcaaagccatcactag<br>cacactttgagatggctagg<br>agggatgcaaaagagatccc<br>caggtgaagacacctacaga<br>agtgtcaccaaccagatgtg<br>ctttcaacccatctcacaaa<br>tattctattttggcagcacg<br>cacatgaggagaggaccaac<br>cagccactacgcagttttac<br>tgttatactcgattccggta

	tactgttttgtgctaagcca<br>gtaggggaaagaataccagg<br>cacagacgctttcctagaat<br>ggatgccacttaagatttca<br>ggccctttagaagacagaaa<br>tatgaggtaagagccagagc<br>ctgtgtacaatgttctgctc<br>taagtccaccgaggcagatg<br>actcaggtatttccttctag<br>taacagggacagcacaccag<br>cagttgtcaccttgacagaa<br>gacacacagatgatgggtca<br>ctagcaaagcatttgccttt<br>caggtatggctttgaacagt<br>ccacagggtttgaagttcaa<br>gagaaagatgccactgaatc<br>aagacaggctgtgtggtgta<br>gtaactcaacagtccacagt<br>tacagatatgcggtgcttac<br>attcaaacacggctaccaca<br>tggttccccaaaaagggaat
14-16K	catgtacacagatatgcggt<br>aatttacacatgctgtgccc<br>gggggtgtccatcatattta<br>ggtgaaggtgtggggaaatg<br>actattttccacttctcaca<br>atcagtaagtatacaaccca<br>ctcacaaaatccaggaacca<br>gctggattagccttttcaaa<br>gttaggagaaaacatggggt<br>aagtctagatagagtcctcc<br>cctgtcacttgttatgctaa<br>ggtgtaaggacaacaggcta<br>gttaatagctgtattagcca<br>aataatgaggtagggcttcc<br>cggcatgaagacatcacagg<br>gacgtaccttattcttgcat<br>aatagacgtgagtggatgga<br>gctttctgtgtaatgcaaag<br>cgaggtagacagaccaagac<br>ggcagtgaggacaactagat<br>atggggtagtcagtcagatg<br><br>ccattaggcagagcaaaacg<br>tcggcgtgacagtatcaagt<br>gcaaagaccactgaactgga<br>tgtgtacgggagcatttcaa<br>acttactggcatttttaaca<br>ggtgcaagctgggtttgtat<br>ctacgctctctgaatgtgac

	tggaaaacaacccatcccag<br>gaagagctagccacacagtg<br>acattccgggtacacagaac<br>gctggacactagaacaggac<br>agcaggaataggctgttgag<br>aaaaagttccagcaggctgt<br>ccagcatggcaacatatttt<br>catgaaaaaggctgccaggg<br>taggacaattacccctttgg<br>aactcatcttacagaccacc<br>atgtctattagcaagtcacc<br>gccactgaatagaacatttc<br>tagtgtcgatggttgcacaa<br>cgtctgtttgggatgacgaa<br>gagacaggtaggaagttatt<br>aggttaccaaagactagggg<br>gcacaacaaagtttaccagt<br>ggcaaattcacacagacaga<br>attacacttatatgaggccc<br>agtgaaatcagacagaccca<br>gaatgaaccctgaaaacccg<br>acgcagtactgttatatgct<br>gtggaatattattcagccag<br>acaaaatggggtctatccat
16-18K	accattgttcataatagcca<br>acttgtacgcaactgttcat<br>attctactcctaggtatata<br>atacagttaccatatgaccc<br>tagtttggcagttcctcaaa<br>gaaatggtgcatggacgtgg<br>ctcttacttactggtgggaa<br>gttggcaagaatgcgcagaa<br>ggctacttcacactcattag<br>tgcaaatccaaatgacacca<br>atagcatcactcatcagaga<br>aacagcaccagcaaaggtgc<br>cagggaacatacacatatgg<br>aggctcagcacagacacttc<br>cgcctaattaaaaagtgggc<br>agggtctagaatccagaata<br>tttgcaaaccatgtatctga<br>caacccacagaatgggagaa<br>cttgcacagccaaatcaagt<br>aagtgctgggattacaagcg<br>acaagttttcgctatgctgg<br>accatgcctgactaattttt<br>cctgagaagctggtattaca<br>ctgggttcaagtgatcctcg<br>atgatctcagctcactgcaa<br>caaagtctcattctgttgcc<br>tcccttaaatcaagtcactt

	ctgactgaatatagccagtc<br>aacaccttgagactttttgc<br>cccaagaccttaatcattca<br><br>tttctgtgcctctaaacacc<br>attaagaacccaactgcacc<br>agttgtgcttctcatcattt<br>tgtctgctgacattctacac<br>gatccagcacatctagcagg<br>acaaatggaaatgctttccc<br>caccacacccatcataaatt<br>cacagtgctgggactacagg<br>ggctgatcttgaactcttgg<br>tagggtatcactatgttgcc<br>tttgcataaatccagtacct<br>ctcatctcaaaagacgcaga<br>gggacttagtattccattaa<br>agcttggttcagaaatcaca<br>aataaaactcaggcctagga<br>gcttggccttctaatttatt<br>actaggactacaggcacatg<br>aagcaatcctcttacctcag<br>agtggcgcaatcaaagcttg<br>tttgagacagcgtcttagtc<br>gttttttttttgtcgttttt<br>ccttagtttattttttctgt<br>catttgccagggactgtcat<br>atttgctttacaagtcctgc<br>ctcccaatcttatttgcttt<br>gaactcctgacctcgtgatc<br>aagacggggtttcactatgt<br>cacctggctaatttttttgt<br>caagtagctgggactacagg<br>ctgggttcgagattctcctg<br>tctcagctgactgtaacctc
18-20K	caggctggagtgcaatggtg<br>ttgagacggaatcttgctct<br>tcttcttggttttttttttt<br>agaagttctaattcattcct<br>atgatgcccttaagtccaag<br>tgctatcctacccaaaacct<br>cacactcatgaccactgacc<br>accaagtttttgggctcaaa<br>actgagtggcaaagtaggga<br>aatttggccaaagtcatgca<br>ccggaaacttgcttcatgaa<br>tttttatccccatttcattc<br>ccaacggataggtttgaaca<br>attcatttcagtttcacaca<br>tcacaagtacctgcacgata<br>agggacaggaaacagcagct

	gtctagatatttcccatcat<br>cagtgcagagggttgggaac<br>cagctggtgttacaagaccc<br>agctcctaaaagaaggccag<br>ccccacatctactaaaacaa<br>cagtccgggcaacacagaaa<br>ggatcacttgagtccagaag<br>aacactttgggaggttgagc<br>agtggctcacacctgtaatc<br>aaaagttttcaaggctgggc<br>atttaatagcccagtaaccc<br>tgctactctacactaataca<br>ctgctttttcaaaaggcca<br>gcagaactgcttactttatc<br>acaaaatccaaggcatgccg<br><br>acaatgcgagcctctagaaa<br>ggcaagcgtgatcataagtg<br>ccctcttccattcttaaaag<br>tagagcaagactgcctcaca<br>caattgaggaaccagcgtgg<br>tcctcggatggcatcagtag<br>gcccacacgaaaccttacat<br>ctctgtggaatgaggcaaca<br>gatgttgatggagtgaccat<br>ctgtcagctgaatgcctgtg<br>agaacagcagcagcgtgaag<br>aatgatttgtgcattgccac<br>ccttggtaactgtcacaact<br>gttagggctgttttcactgg<br>cttcgcagacaggaatgctg<br>cagcttgcatgacttcaggc<br>tcacgtcctgaatcctcatg<br>tacagatggaaggccttcat
20-22K	cacttcctcagataaccatt<br>tcaatgaagaaagggagcca<br>atgtgacagagaatcccttc<br>aatggggaggaactgatcca<br>gcagatcggatttgaccaac<br>ctcacttgtcattacacagg<br>aggagtgacggtgagaatgc<br>ctcaggggaagcggagagac<br>catggagcagactgctgagc<br>tgggtcaccctgcatcaaaa<br>ggcgttagtgtcgggaatac<br>agacacagtgaagcccaagc<br>ttaggtctgcactgtcaaca<br>gtccagtgcaaacaagaaga<br>gcagttgaaaataaccctca<br>ccacatagtaactaccaatc<br>ttcagcccaagtaattgtgt

	ccaaccacaacaggtggatt<br>aaccacaaaatgctggtgcc<br>tagaaggggatttgcccaaa<br>ggttgcaaaccaaaagctgc<br><br>gagctacgcggggaaacgtg<br>gggtgtgtgctgtgggataa<br>ttcagcgtgttagcaacaga<br>gggcagtcaaaacacactct<br>ccttacaaggcctcagaaat<br>gccgatgaagcaacaaagct<br>cattcacggccatctaagtc<br>aaacacactatggtgcgggc<br>gaatgtgttgggccaactgc<br>tcagacctctcaaaggggtg<br>gaatctgagcagaatcagcc<br>agtgctaaggagctcagaag<br>caggacccaaaggtacacaa<br>tcctgtcacttgactctaac

References

1. Guo, S.-M. *et al.* Multiplexed and high-throughput neuronal fluorescence imaging with diffusible probes. *Nature Communications* **10**, 1–14 (2019).

2. Rieger, B., Droste, I., Gerritsma, F., Ten Brink, T. & Stallinga, S. Single image Fourier ring correlation. *Opt. Express* **32**, 21767–21782 (2024).
