# Supplementary material for: Smart 3D super-resolution microscopy reveals the architecture of the RNA scaffold in a nuclear body
Source: Nat Commun. 2025 Nov 27;16:10689. doi: 10.1038/s41467-025-65723-x (PMC12660752; doi:10.1038/s41467-025-65723-x)
Supplement: Supplementary file 3 — Description of Additional Supplementary Files [file 41467_2025_65723_MOESM3_ESM.pdf]

Supplementary Movie 1: Shell-stained paraspeckles (bounding box sorting)

- This movie shows all rotationally and translationally aligned paraspeckle images, sorted by their length as calculated from the 50% intensity drop-off point. On the left, we show a 3D reconstruction composed of 5 isosurfaces from 50% to 99% of the maximum image intensity. On the right, we show the 3 cross-sections through the image center.

Supplementary Movie 2: Averaged Shell-stained paraspeckles (bounding box sorting)

- This movie shows the bin-averaged paraspeckles. On the left, we show a 3D reconstruction composed of 5 isosurfaces from 50% to 99% of the maximum image intensity. On the right, we show the 3 cross-sections through the image center.

Supplementary Movie 3: Averaged Shell-stained out of ROI paraspeckles (covariance sorting)

- This movie shows the bin-averaged averaged out-of-ROI paraspeckles. On the left, we show a 3D reconstruction composed of 5 isosurfaces from 50% to 99% of the maximum image intensity. On the right, we show the 3 cross-sections through the image center.

Supplementary Movie 4: showing all 3 /5' stained paraspeckles with ellipticity ≤ 1.3. Green: 5' signal; magenta: 3' signal.

- This movie shows isosurfaces of the signal from single spherical paraspeckles (ellipticity ≤ 1.3) stained at the 5'-end (green) and the 3'-end (magenta) of NEAT1_2. The paraspeckles were rotated to align the polarization vector with the vertical axis of the image frame and the images were sorted by ascending degree of polarization. The degree of polarization is indicated above each isosurface.

Supplementary Movie 5: showing all 3 /5' stained paraspeckles with ellipticity > 1.3. Green: 5' signal; magenta: 3' signal.

- This movie shows isosurfaces of the signal from single elongated paraspeckles (ellipticity > 1.3) stained at the 5'-end (green) and the 3'-end (magenta) of NEAT1_2. The paraspeckles were rotated to align the polarization vector with the vertical axis of the image frame and the images were sorted by ascending degree of polarization. The degree of polarization is indicated above each isosurface.
